# A Global Review of Food-Based Dietary Guidelines

**DOI:** 10.1093/advances/nmy130

**Published:** 2019-04-30

**Authors:** Anna Herforth, Mary Arimond, Cristina Álvarez-Sánchez, Jennifer Coates, Karin Christianson, Ellen Muehlhoff

**Affiliations:** 1 Intake, Center for Dietary Assessment, FHI 360, Washington, DC; 2 FAO Statistics Division, Rome, Italy; 3 Tufts University Friedman School of Nutrition Science and Policy, Boston, MA; 4 FAO Regional Office for Africa, Division of Partnerships and South-South Cooperation, Accra, Ghana; 5 Independent

**Keywords:** food-based dietary guidelines, dietary recommendations, recommended diet, healthy diet, nutritious diet

## Abstract

The objective of this review is to provide a concise, descriptive global review of current food-based dietary guidelines (FBDG), and to assess similarities and differences in key elements of a healthy diet articulated across countries. Information was sourced from the FBDG repository of the FAO, which catalogs FBDG for all countries where they are available, including a description of the food guide (the graphic representation of the dietary guidelines), a set of key messages, and downloadable documents provided by the countries. FBDG are currently available for 90 countries globally: 7 in Africa, 17 in Asia and the Pacific, 33 in Europe, 27 in Latin America and the Caribbean, 4 in the Near East, and 2 in North America. The year of publication of current versions ranges from 1986 to 2017 (mean 2009). This review provides summaries of the key messages and food guides that are used to communicate national dietary guidance, organized by food group, and evaluates the extent to which each set of FBDG includes existing recommendations articulated by the WHO. Some guidance appears nearly universally across countries: to consume a variety of foods; to consume some foods in higher proportion than others; to consume fruits and vegetables, legumes, and animal-source foods; and to limit sugar, fat, and salt. Guidelines on dairy, red meat, fats and oils, and nuts are more variable. Although WHO global guidance encourages consumption of nuts, whole grains, and healthy fats, these messages are not universally echoed across countries. Future frontiers in FBDG development include the incorporation of environmental sustainability and increased attention to sociocultural factors including rapidly changing dietary trends. Steps toward regional and global dietary recommendations could be helpful for refinement of country-level FBDG, and for clear communication and measurement of diet quality both nationally and globally.

## Introduction

Food-based dietary guidelines (FBDG) are an attempt to translate a vast (and always incomplete) evidence base regarding relations between foods, diet patterns, and health into specific, culturally appropriate, and actionable recommendations. Such guidelines are intended to influence consumer behavior and, in some countries, also inform a range of national food, nutrition, and health policies and programs.

Development of FBDG is both a scientific and political process, incorporating a range of evidence and stakeholder perspectives (1). The types of evidence used to inform FBDG include: assessments of food and nutrient intakes, food supplies, prevalence and public health importance of diet-related health and nutrition outcomes, cultural preferences, and other considerations. Since the 1998 publication of *Preparation and use of food-based dietary guidelines* based on a joint WHO/FAO consultation (2), many countries and regions have developed their own national guidelines, often in partnership with, or facilitated by, international agencies and bodies. Some regions have developed regional recommendations, such as the Nordic Nutrition Recommendations, which are the main basis for national FBDG in Denmark, Finland, Iceland, Norway, and Sweden (3). There has been coordination on guidance for developing FBDG in Latin America and the Caribbean (4, 5), and in the Western Pacific region (6).

Shaped by their context, national- or regional-level FBDG vary widely in their specifics. However, there are many recurring elements and themes (1, 7).

There have been several attempts to summarize FBDG, including globally (8); regionally for Europe (9–11), North America (12), Latin America and the Caribbean (12, 13), the Spanish-speaking Caribbean (14), and Southeast Asia (15); in selected countries (16); and for the specific purpose of examining sustainability in FBDG (17). The recent global review of FBDG by van't Erve et al. (8) is the only other examination of all current FBDG globally, and focuses on a description of which food groups are included and graphic design aspects, with the key concern of effectiveness and use by consumers. To our knowledge, no comprehensive global review to date has analyzed key messages, level of concordance and difference across countries in key messages or graphics, or the comparison of national FBDG with global dietary recommendations. In 2014, the FAO completed a major update and website launch for their online repository for FBDG and associated resources (18). This resource is continually updated, and opens a new opportunity to comprehensively review the state of FBDG globally. Using the FAO FBDG repository, this review is able to present in-depth descriptions and comparisons of countries’ key messages and graphics. Further, the Second International Conference on Nutrition Framework for Action (19) recommended the development of international guidelines on healthy diets (Recommendation no. 13). This assessment of commonalities and differences across existing national guidelines is useful to inform the acceptability of international guidelines that might be considered.

## Objective and Scope

The objective of this article is to provide a concise, descriptive global review of current FBDG. The purpose is to assess the level and type of concordance and differences across countries’ existing guidance on key elements of a healthy diet. We provide summaries of the key messages and graphics that are used to communicate national dietary guidance. Further, the review evaluates the extent to which each set of FBDG addresses existing recommendations articulated by WHO in the Healthy Diet Fact Sheet (20).

This review does not: summarize guidance for specific population subgroups (e.g., young children, pregnant and lactating women, and the elderly); examine the range of evidence used in the process of developing each FBDG; examine the process and range/type of stakeholders engaged in development; evaluate impacts of FBDG on consumption behavior; or examine detailed guidance documents, many of which are only available in national languages.

## Methods

The information source for this review was the FAO FBDG repository (18), which collates information gathered through a 2013–2014 global survey on FBDG development, use, and evaluation, and has subsequently been continually updated. Country pages usually include, in English: the official name of the national guidelines; publication year; the intended audience or target group for guidelines; a description of the food guide (the graphic representation of the dietary guidelines); and a set of key messages (brief statements written in text) provided by the responsible country authorities. Each page also usually includes the food guide itself and links to downloadable documents, including full guidelines and any available communication resources provided by the countries. All materials on the website have been sourced and verified by the responsible government agency at country level.

For this review, information was extracted from the webpages in September, 2017, and updated in July, 2018 to reflect additions. Webpages were available for 90 countries: 7 from the Africa region, 17 from the Asia and the Pacific region, 4 from the Near East region, 33 from the European region, 27 from the Latin America and Caribbean region, and 2 from North America. Our analysis of FBDG covers key messages, food guides, and other brief consumer-facing guidance intended for the general public. Longer documents provided on the same webpages were consulted when necessary to determine country-specific food group definitions when food guides were unclear.

Verbatim key messages were organized by food groups commonly present in food guides: starchy staples, vegetables and fruits, various protein foods (including meat, poultry, fish, eggs, legumes, nuts, and seeds), dairy, fats and oils, and foods and food components to limit. Within these major food groupings, analysis of key messages generated themes through an iterative and inductive process. Messages that articulated 2 food groups in 1 sentence were cross-classified under both groups. Messages were classified as qualitative or quantitative: quantitative messages stated specific guidance on the quantity (grams, servings) and/or frequency (number of times per day or week) that foods or food groups should be consumed. The frequency and type of key message for each food group, and the elements included in food guides, were summarized globally and by region. Key messages and food guides were also analyzed for their relation to dietary recommendations from the WHO Healthy Diet Fact Sheet (20)—which is based on the WHO Global Strategy on Diet, Physical Activity, and Health (21) and the WHO/FAO Expert Consultation on Diet, Nutrition and the Prevention of Chronic Diseases (22). In a similar manner to national FBDG, the WHO Healthy Diet Fact Sheet includes guidance both on whole foods and on salt, sugar, and fats, which can be either foods (table salt, table sugar, oil/butter) or food components (salt, sugar, and fat content of prepared foods).

## Results

### Overall description of FBDG

**Table 1** shows the number of countries that have FBDG, mean year of publication, and version globally and by FAO-defined region. The average year of publication of current FBDG is 2009. **Table 2** shows a timeline of countries by year of FBDG publication. UN organizations—WHO/Pan American Health Organization (PAHO), FAO—provided technical assistance in the development of FBDG in 42% of countries. All FBDG state that they apply to the general population; 46% qualify this statement with “healthy” population; 13% refer to the general “adult” population. Over half (52%) specify an age above which the guidelines apply: almost all of these refer to age 2 y and older, although 2 countries instead specify age 1 y, 1 country says age 3 y, and 3 say age 5 y. Forty percent of countries have separate messages or guidelines for specific subpopulations (not analyzed here), which include infants under age 2 y, school-aged children, adolescents, pregnant and lactating women, the elderly, and others.

**FIGURE 1:**
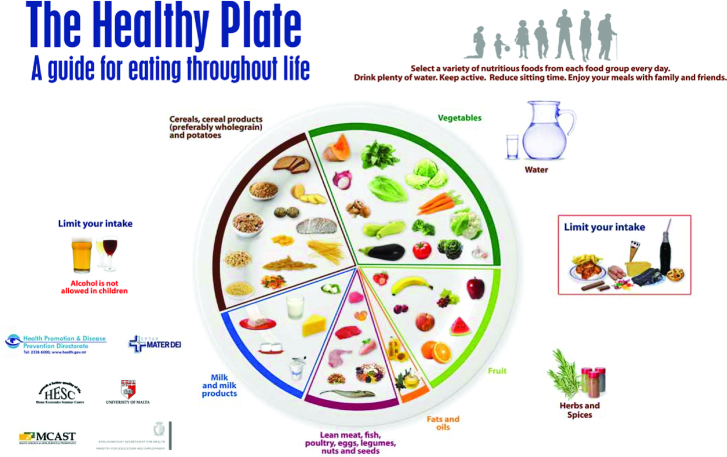
Malta: a plate-style food guide. Reproduced from reference 23 with permission.

**TABLE 1:** Existence of guidelines, year of publication, and version, by FAO region and at global level

	Global	Africa	Asia and Pacific	Europe	Latin America and Caribbean	Near East	North America
With any FBDG, *n*	90	7	17	33	27	4	2
With a food guide, *n* (%)	78 (87%)	6 (86%)	15 (88%)	27 (82%)	24 (89%)	4 (100%)	2 (100%)
Year of publication (mean)^1^	2009	2010	2011	2008	2010	2011	2012
First FBDG, *n* (%)	41 (46%)	6 (86%)	1 (6%)	17 (52%)	14 (52%)	3 (75%)	0 (0%)
If not first, years since revision (mean)^1^	10	9	11	11	10	10	8
Stated plan to update FBDG within the next few years	13 (14%)	0	2 (12%)	9 (27%)	0	0	2 (100%)
UN (WHO/PAHO, and/or FAO) collaborated in development, *n*(%)^1^	38 (42%)	6 (86%)	5 (29%)	7 (21%)^2^	18 (67%)^3^	2 (50%)	0 (0%)

^1^Rounded to the nearest unit.

^2^Apart from UN involvement, 5 European countries base their recommendations on the Nordic Nutrition Recommendations (3).

^3^Apart from UN involvement, 15 of 27 (56%) Latin America and Caribbean (LAC) countries involved a regional nutrition/health organization such as Instituto de Nutrición de Centroamérica y Panamá or the Caribbean Food and Nutrition Institute; in total, 78% of LAC countries involved the UN *and/or*a regional organization. PAHO: Pan American Health Organization.

**TABLE 2:** Publication timeline of current food-based dietary guidelines (FBDG)

Before 2000	2000–2004	2005–2009	2010–2014	2015–2017
Greece	Bahamas	Albania	Antigua and Barbuda	Afghanistan
Malta	Bosnia and Herzegovina	Barbados	Australia	Argentina
Thailand	Croatia	Belgium	Austria	Benin
Venezuela	Guyana	Bulgaria	Bangladesh	Indonesia
—	Hungary	Canada	Belize	Jamaica
—	Italy	China	Bolivia	Kenya
—	Namibia	Cuba	Brazil	Mexico
—	Nigeria	Cyprus	Chile	Paraguay
—	Portugal	Dominica	Colombia	Qatar
—	Turkey	Dominican Republic	Costa Rica	Seychelles
—	—	Estonia	Denmark	Sierra Leone
—	—	Fiji	El Salvador	Sweden
—	—	Georgia	Finland	United Kingdom
—	—	Grenada	France	United States of America
—	—	Iceland	Germany	Uruguay
—	—	Iran	Guatemala	—
—	—	Israel	Honduras	—
—	—	Latvia	India	—
—	—	Oman	Indonesia	—
—	—	Romania	Ireland	—
—	—	Saint Lucia	Japan	—
—	—	Saint Vincent and the Grenadines	Lebanon	—
—	—	Spain	The former Yugoslav Republic of Macedonia	—
—	—	—	Malaysia	—
—	—	—	Mongolia	—
—	—	—	Nepal	—
—	—	—	Netherlands	—
—	—	—	Norway	—
—	—	—	Panama	—
—	—	—	Philippines	—
—	—	—	Poland	—
—	—	—	Democratic People's Republic of Korea	—
—	—	—	Saint Kitts and Nevis	—
—	—	—	Slovenia	—
—	—	—	South Africa	—
—	—	—	Sri Lanka	—
—	—	—	Switzerland	—
—	—	—	Vietnam	—

### Description of food guides

**FIGURE 2:**
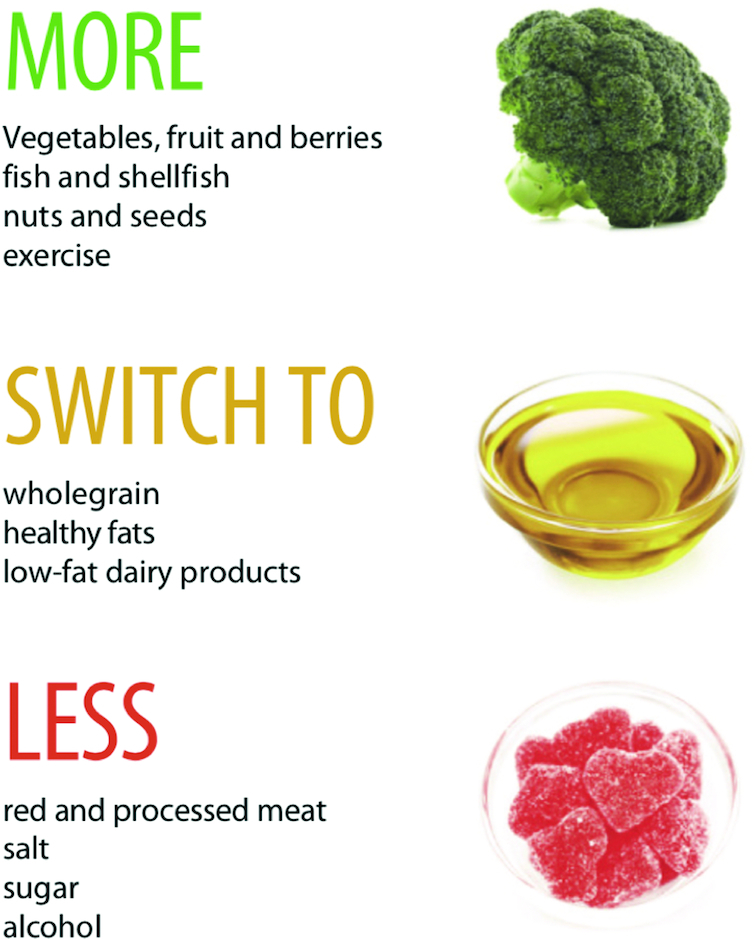
Sweden: an abbreviated food guide. Reproduced from reference 24 with permission.

Most countries with FBDG (87%) publish a food guide, the official term for a graphic representation of the guidelines. Food guides are intended to provide dietary guidance to the general public by conveying through pictorial images the concepts of variety, proportionality, and adequacy/moderation to meet population dietary needs. Among the 78 countries with food guides, with very few exceptions they include various food groups, usually illustrated with photographs or drawings of numerous example foods in each group. Rarely (e.g., Greece, Hungary), the food groups are identified by text only, with no example items shown. The majority are pyramids, plates, or cultural shapes, such as a basket, house, or pineapple. A few are other shapes, such as a diagram of individual plates or groups. Previous published work provides an extensive description of the graphical elements of countries' food guides (8). **Table 3** summarizes some characteristics of the food guides. Two examples of food guides are shown in **Figures 1** and **2**.

**TABLE 3:** Elements included in food guides

	Number	% (of 78)^1^
Conveys proportionality between food groups^2^	74	94.9
Includes recommended quantity or number of servings	27	34.6
Shape of food guide		
Pyramid or inverted pyramid	31	39.7
Circle or plate	21	26.9
Cultural item (e.g., basket, pot, drum, mortar, map, nutmeg, sugar mill)	22	28.2
Other (diagram, rainbow, stoplight)	7	9.0
Number of food groups illustrated, excluding fats/oils and sweets/sugars^3^		
Three	9	11.5
Four	21	26.9
Five	40	51.3
Six or more	8	10.3
Includes fats/oils^4^	68	87.2
Includes sweets/sugars^4^	55	70.5
Includes other items for moderation^4^, ^5^	28	35.9
Includes exercise^4^	38	48.7
Includes water^4^	44	56.4

^1^Denominator is the 78 countries that use a food guide and provided it to the FAO.

^2^A food guide is considered to convey proportionality if food groups are shown in different sizes. Note that the nature of the proportionality messages (balance between groups) varied considerably.

^3^To identify the number of groups, both the food guide and the verbal description of the food guide provided by country authorities to the FAO and included on country webpages were examined. In cases where there was ambiguity, text provided by the country authorities was taken first; in the absence of explicit text on groups, groups were considered separate if separate group names or portion recommendations were included on the graphic. Because 15–20% of food guides do not include either fats/oils or sweets/sugars, and because these groups usually are recommended in moderation, they are excluded from this count, despite that in some countries there is a “healthy fats” group that is encouraged. The remaining food groups are usually encouraged for daily consumption; in some food guides, some food groups are encouraged for less frequent consumption (e.g., X times per week for meat or fish).

^4^Food guides were considered to “include” the group or element if there was a graphic image or text integrated into the graphic itself. Key messages provided in lists below the graphic on a few guides are not included.

^5^Usually, the other items include depiction of “fast food” and/or processed meats (fried potatoes, hamburgers, sausage).

The concept of variety is embodied in all food guides. Nearly all (95%) convey the concept of proportionality, namely that different food groups should be consumed in differing amounts. Most graphics convey the concept of moderating or limiting consumption of some food types, such as sugars/sweets and fats/oils. Excluding fats/oils and sugars/sweets, more than half of countries encourage consumption of 5 food groups, with the most common set of 5 groups being: starchy staples (variously defined); fruits; vegetables; dairy foods; and other “protein foods” (also variously defined). The most common 4-group combination is: starchy staples; fruits and vegetables; dairy; and other “protein foods.” The most common 3-group combination is: starchy staples; fruits and vegetables; and “protein foods.” There is much consistency in the Latin America/Caribbean (LAC) region, with most countries identifying 5 groups (starchy staples; fruits; vegetables; legumes; and animal-source foods [ASF]). There is wide variability in Europe; several Mediterranean countries present a larger number of food groups, including, for example, olive oil, fish, and nuts as separate food groups.

Globally, there is wide variability in the way foods are categorized, particularly for legumes, nuts, and ASF, and, to a lesser extent, for fats, oils, and oilseeds. Groupings for fruits and vegetables are relatively consistent, but there are some inconsistencies in classifications of potatoes and other roots and tubers, legumes, and fruit juice. The FAO/WHO FBDG expert consultation report (2) does not make any recommendation on how to group foods, acknowledging that countries group foods differently. The next sections provide details on classification of these food groups, and also provide analyses of key messages related to food groups.

### Starchy staples

All countries with food guides include starchy staples in their food guide, but not all include them in their key messages; 66% of countries include key messages concerning starchy staples, and an additional 16% imply their consumption within a general dietary variety message that refers to the food guide or graphic. Few are quantitative, with 14% of countries providing a quantitative recommendation for either starches as a whole (e.g., *“*Eat cereal-based foods three times a day”: Sri Lanka), or whole grains specifically (e.g., “Replace rice with wholegrains and other high-fibre starchy foods at least 3 times a week”: Seychelles). Consuming starchy staples daily or as part of most meals is advised by 27% of countries; 11% recommend consuming “plenty,” “mainly,” or “more” (“Eat plenty of cereals, preferably wholegrain, and potatoes”: Germany), whereas 6% recommend consuming “adequate,” “enough,” or “appropriate” amounts (e.g., “Eat enough grains such as rice and other cereals”: Japan).

In one-quarter of countries with a key message about starchy staples, the message is exclusively about whole grains. Whole grains are mentioned explicitly by 44% of all countries, mostly in Europe (70%), North America (100%), and the Near East (75%). Ten percent of countries have key messages mentioning fiber, principally in relation to whole grains.

Many countries (29%) have key messages concerning starchy roots and tubers, such as potato and/or cassava, together with grains in a starchy staple message. There is regional variation, with a higher probability of a roots/tubers mention in Africa (57% of countries with FBDG) and in Europe (42%).

In food guides, starchy staples are portrayed visually as the largest group in 59% of countries, whereas fruits and vegetables combined are the same size or bigger in 40% of countries. Starchy staples are more likely to be the largest group in the Near East (100%) and Africa (83%), and less likely in Europe (37%) and North America (0%). About half of countries show a clear graphical representation of whole grains (53%), most often in Europe. Most food guides (65%) include starchy tubers and/or plantains in the starchy staple group along with grains.

### Fruits and vegetables

Considering the food guides as well as key messages, all countries encourage consumption of fruits and vegetables. There are key messages specifically about fruits and vegetables in 93% of countries (**Table 4**). Many countries convey multiple messages aimed to encourage consumption of fruits and vegetables. Some messages are simple and cover 1 dimension (see examples in Table 4) but others are multidimensional, such as “Eat various types of vegetables and fruits several times a day (at least 400 g/day) preferably fresh and locally produced” (Albania).

**TABLE 4:** Key messages about fruits and vegetables conveyed by more than 5 countries—types and examples

	Number^1^	% (of 90)
Any key message about fruits and vegetables	84	93.3
Eat daily (or with every meal)	62	68.9
“Eat plenty of vegetables and fruit every day” (South Africa)	—	—
Five (or more) servings a day, or 400 g (or more)	30	33.3
“Consume three or more servings of vegetables and at least two servings of fruit per day” (Mongolia)	—	—
“Eat vegetables, fruits and berries frequently (a minimum of 500 g/day, excluding potatoes)” (Finland)	—	—
Variety within	38	42.2
“Choose a variety of fruits and vegetables every day” (Bahamas)	—	—
Eat plenty or “a lot”	19	21.1
“Consume plenty of vegetables, fruits and tubers” (China)	—	—
Eat more	16	17.8
“Increase your consumption of fruits and vegetables. Eat five portions of fruits and vegetables a day” (Cyprus)	—	—
Eat different colors or particular colors	17	18.9
“Eat at least one dark green and one orange vegetable each day. Go for orange vegetables such as carrots, sweet potatoes and winter squash. Go for dark green vegetables such as broccoli, romaine lettuce and spinach” (Canada)	—	—
“Eat five fruits and vegetables of different colours and flavours every day to fill you with health and vitality” (Dominican Republic)	—	—
“Eat plenty of green leafy vegetables, red and yellow vegetables and fruits every day; and include a variety of other vegetables and fruit” (Kenya)	—	—
Special mention/emphasis on whole, raw, or unprocessed	10	11.1
“Eat more than 400 g of various fruits and vegetables every day. Eat some of them raw” (FYROM)	—	—
Eat fresh fruits/vegetables	9	10.0
“Increase your daily intake of fresh fruits and vegetables of different colors” (Panama)	—	—
Eat seasonal fruits/vegetables	6	6.7
“Prefer vegetables and fruits that are locally grown in season. Whenever possible, buy organic and agro-ecological based foods, preferably directly from the producers” (Brazil 2014)	—	—
Eat local fruits/vegetables	7	7.8
“Eat more local fruits and vegetables” (Fiji)	—	—
Not mentioned but implied in diversity message (by reference to food guide)^2^	3	3.3
“Eat foods from each food group every day to have a complete diet” (Portugal)	—	—
No key message conveyed but fruits and vegetables are shown in food guide	6	6.7

^1^Sums to more than 90 because some countries have multiple messages about fruits and vegetables. Also many key messages contain more than 1 idea and are counted for several. For example, “Eat five servings (approx. 400 g) of vegetables, fruits and berries every day. Try to choose local and fresh products” [Latvia] is counted under “Eat daily,” “Five (or more) servings,” “Fresh,” “Local,” and “400 g/day (or more).”

^2^Considered to be implied when a key message directs the reader to eat all food groups, and fruits and vegetables are separate food group(s) in the food guide (this is so in nearly all food guides).

The most common key messages concerning fruits and vegetables are to eat them daily (69% of countries); to consume a variety (42%); and to eat “plenty,” “a lot,” or “more” (38%). WHO guidance is to eat at least 400 g (5 portions) of fruits and vegetables per day (20). One-third of countries recommend either 5 servings or more per day, or 400 g/d in their key messages; roughly half (51%) of countries give this quantitative recommendation through either key messages *or* food guides. Guidance to choose a variety of colors or particular colors is found in 19% of countries, with 10% of countries recommending consumption of green leafy vegetables, and 6% specifically recommending orange fruits and vegetables. Four countries (all in Europe) make a special mention of berries.

Less common messages are to consume fruits and vegetables that are fresh (10% of countries), local (8%), or seasonal (7%). Special mention of whole, raw, or unprocessed forms of fruits and vegetables is made by 11% of countries, whereas only 3% of countries note that fruits and vegetables can be consumed in various forms (e.g., cooked, part of sauce, etc.). Fruit and vegetable consumption is urged in terms of vitamin and mineral content in 8% of countries, for fiber content in 7% of countries, and for maintaining a healthy weight or preventing disease in 3% of countries.

There is some regional variation in messages, with quantitative messages citing amounts in grams as a key message expressed only by European countries (21% of European countries) and conveyed by graphics or in brief written guides for 2 Asian countries. The key message to eat fruits and vegetables daily is less often stated by Asian countries (35%) compared with several other regions (Africa 71%; Europe 79%; Latin America/Caribbean 81%). The message on variety within fruits and vegetables is more likely to be conveyed in North America (both countries) and in Latin America/Caribbean (59% of countries) compared with other regions (36% in Europe, 29% in Asia, and only 1 of the African guidelines).

**Table 5** summarizes how fruits and vegetables are shown in food guides. In the majority of countries where it could be determined, fruits and vegetables are pictured and/or described as separate groups. However, in a significant minority they are grouped together. This distinction is somewhat artificial, because, for example, in pyramid or inverted pyramid graphics, fruits and vegetables are often 1 “layer” with fruits on one side and vegetables on the other, and with or without a line between the two.

**TABLE 5:** Grouping of fruits and vegetables in food guides

	Number	% (of 78)^1^
Fruits and vegetables grouped together	33	42.3
Fruits and vegetables comprise 2 groups	45	57.7
Potatoes included with vegetables^2^	14	17.9
Inclusion of potatoes cannot be determined^3^	3	3.8
Legumes included with vegetables	9	11.5
Inclusion of legumes cannot be determined^3^	6	7.7
Juice included	18	23.1
Inclusion of juice cannot be determined^3^	30	38.5

^1^Denominator is the 78 countries that use a food guide and provided it to the FAO.

^2^In most cases, Irish potatoes are the only pale root/tuber depicted with vegetables; in the very few cases where other starchy pale roots/tubers (e.g., yams) are included with vegetables, Irish potato is also included. Other colored root vegetables (beetroot, carrot, etc.) are consistently included with vegetables, as are turnips, radishes, etc.

^3^Groupings could not be determined when: *1*) the food item was not clearly pictured, or when the food guide was scanned and quality was poor; and *2*) when other available documents either could not be translated or did not mention the food items in group descriptions or portion size examples.

Guidance on the consumption of fruit juice is mixed globally, and sometimes across documents within countries. Relatively few countries depict fruit juice visually as belonging in the fruit group on food guides, but a larger number of countries include fruit juice in the description of the fruit group or in portion size examples for the fruit group. Many countries include fruit juice but at the same time deliver messages on moderation in longer guidance, stating that fruit juice should not count for more than 1 serving per day of fruit, or that whole fruits should be preferred to fruit juice. For example, the South African dietary guidelines state that juice is “acceptable as an occasional substitute” (25, p. 165). A few countries (7%) explicitly deliver negative messages on fruit juice and/or group fruit juice with other sweet beverages.

In food guides, most countries exclude potatoes and other pale starchy roots and tubers from the vegetables group and group them with starchy staples. In a few countries, it is explicitly noted that although potatoes do not count toward the WHO target for consumption of fruits and vegetables, they are nevertheless part of a healthy diet (e.g., Sweden, Norway) and/or are culturally important (e.g., New Zealand). Legumes are more commonly grouped with protein foods or in a separate group. They are more likely to be grouped with vegetables in Europe; outside of Europe, only 3 countries grouped legumes with vegetables. Among countries where grouping was clear both for potatoes and for legumes (*n* = 70), 73% excluded both from the vegetables group.

Starchy fruits such as plantains or green bananas are pictured or mentioned only in regions and countries where commonly consumed, including the Caribbean, Central America, several countries in Africa, and Fiji. With the exception of 2 countries (El Salvador and Sri Lanka), they are grouped with starchy staples.

### Protein foods

Countries discuss and display what can generally be termed “protein foods” in a variety of ways. This section does not discuss country messages and depiction of dairy, which in most countries is treated as a separate food group. We include animal and plant-source proteins in this general category in order to summarize the variety of ways countries have categorized them, while recognizing that nutritionally, animal and plant sources of protein are not equivalent in terms of amino acids, fatty acids, vitamin B-12, bioavailable iron and zinc, fiber, and plant secondary metabolites.

Although not all FBDG use the word “protein,” 74% include a key message about protein foods, which could include meat (53% of countries), poultry (29%), fish (58%), eggs (31%), legumes (41%), and sometimes dairy (9%), nuts/seeds (8%), and insects (only Kenya) (**Table 6**). An additional 11% of countries do not have a protein foods key message per se, but encourage consumption within a general diversity message that refers to the food groups shown in the food guide.

**TABLE 6:** Key messages about protein foods conveyed by more than 5 countries—types and examples

	Number^1^	% (of 90)
Any key message about protein foods	67	74
“Consume fish, lean meat, poultry, eggs, dried beans or nuts daily for growth and repair of body tissues” (Philippines)	—	—
Mentions both animal and plant sources of protein	33	37
“Eat fish, lean meat, eggs, legumes and pulses regularly” (Thailand)	—	—
Mentions only animal sources of protein	27	33
“Eat either fish, poultry, meat, milk or eggs every day” (Sierra Leone)	—	—
Mentions only plant sources of protein	6	7
“Include peas, beans and nuts in your daily meals” (Jamaica)	—	—
Choose lean meats, or remove fat from meat	31	34
“Choose lean meats (e.g. poultry, rabbit) and fish over red meat” (Croatia)	—	—
“Select lean meat and alternatives prepared with little or no added fat or salt. Trim the visible fat from meats. Remove the skin from poultry. Use cooking methods such as roasting, baking or poaching that require little or no added fat. If you eat luncheon meats, sausages or pre-packaged meats, choose those lower in salt (sodium) and fat” (Canada)	—	—
Limit or moderate meat consumption in general	12	13
“Eat meat in moderation” (Poland)	—	—
“Limit consumption of meat products” (Slovenia)	—	—
Limit or moderate consumption of specific types of meat (red, processed, and/or cured)	10	11
“Eat less red and processed meat, no more than 500 grams a week. Only a small amount of this should be processed meat” (Sweden)	—	—
Limit or moderate egg consumption	6	7
“Eat milk and dairy products every day; fish once or twice a week; and meat, sausages and eggs in moderation” (Germany)	—	—
Eat fish	24	27
“Eat fish at least twice a week” (Iceland)	—	—
“Eat more fish; it's a good source of protein as well as containing important vitamins and minerals. Try to eat oily fish at least once a week, for example, mackerel, sardines and salmon. These are high in omega 3 fats” (Ireland)	—	—
Eat meat	5	6
“To prevent anaemia, schoolchildren, adolescents and young women should eat offal once per week” (Colombia)	—	—
Eat X times per week	34	38
“Eat beans, peas, lentils, cowpeas, pigeon peas, soya, nuts and edible seeds regularly (at least four times a week). Eat lean meat, fish and seafood, poultry, insects or eggs at least twice a week” (Kenya)	—	—
Not mentioned but implied in diversity message (by reference to food guide)^2^	10	11.1
“Eat foods from each food group every day to have a complete diet” (Portugal)	—	—
No key message conveyed but protein foods are shown in food guide	11	12.2

^1^Sums to more than 90 because some countries have multiple messages about protein foods. Also many key messages contain more than 1 idea and are counted for several.

^2^Considered to be implied when a key messages directs the reader to eat all food groups, and protein foods are separate food group(s) in the food guide (this is so in nearly all food guides).

Half of all countries with protein messages (34 of 67 countries; 38% of all countries) include a quantitative message, for example, “Eat five or six servings of fish a week” (Greece). Almost all of these refer to times or servings per week; note that the meaning of a “serving” is not always well defined. Of these quantitative messages, the mean amounts recommended are:

- Pulses/vegetable protein (*n* = 12): 6 times or servings per week (mode = 7 per week).
- Meat/egg/poultry/ASF (variously defined) (*n* = 15): 5 times or servings per week (mode = 7 per week). Of the 15 countries with quantitative recommendations, 11 specified it as a target, and 4 as a limit/maximum. The amounts quantified did not significantly differ whether they were implied as a limit or a target.
- Fish (*n* = 18): 2.2 times or servings per week (mode = 2 per week).
- Red meat (*n* = 3) only appeared in terms of an upper limit amount, which was stated in 2 countries as <500 g/wk (Finland and Sweden), and in 1 country (Greece) as 4 times per month.

The most common themes within key messages about protein foods include: *1*) use of the term “lean meat” or suggesting removing fat from meat (34%); *2*) a positive message about consuming fish (27%); and *3*) limiting or moderating meat consumption (23%). These themes are all particularly common in Europe, where a common theme recurs in several countries’ protein messages, which can be summarized as “eat less meat and more fish.” Examples include: “Choose lean meat, replace meat and meat products often with fish, poultry or pulses” (Bulgaria); “More seafood—Eat fish and shellfish two to three times a week. Vary your intake of fatty and low-fat varieties, and choose ecolabelled seafood. Less red and processed meat—Eat less red and processed meat, no more than 500 grams a week. Only a small amount of this should be processed meat” (Sweden).

Regarding specific types of protein foods, fish appears most frequently in key messages. More than one-quarter (27%) of countries have a special message about fish that is positive (e.g., “Eat more fish”: Denmark), and in 17% of countries, key messages imply that fish is not substitutable (“To keep your heart healthy, eat baked or grilled fish twice a week”: Chile). Only 2 countries include a moderation/limit message around fish: Canada by including advice about limiting mercury exposure from fish, and Belgium by grouping fish together with other ASF to limit.

Half of countries with protein food key messages (33 of 67) include both animal and plant sources of protein. Only 5 countries (all in Latin America and the Caribbean [LAC]) imply that meat is nonsubstitutable, on the basis of providing iron/preventing anemia (“Eat a piece of meat, chicken, liver or fish at least twice a week to avoid anaemia and malnutrition”: Guatemala). In one-third of all countries with protein food messages (23 of 67), non-ASF are presented as substitutes for ASF, through inclusion in the same sentence or explicit substitution messages—for example, “Eat high-protein foods (animal or vegetable source)” (Indonesia); “When there is no meat, fish or eggs in a given day, you can replace them with pulses, peanuts, soybeans, soya, cheese or peas. All these foods are rich sources of protein” (Benin). There are regional differences: in the Asia Pacific region, 9 of 11 countries with protein food messages listed vegetable protein and ASF in the same general message, thus implying that they are alternatives (“Include lean meat, poultry, seafood, eggs or alternatives”: New Zealand), as did both North American countries. However, no countries in the Near East or LAC mention plant-based protein as a substitute for ASF.

ASF are depicted in 100% of food guides. In 54% of countries, food guides depict at least 1 protein group with only ASF (although legumes, with or without nuts, can also be another distinct food group in these countries). In 31% of countries, particularly in LAC, this ASF group includes dairy, whereas in most countries dairy is considered a separate food group. In 44% of countries, protein foods encompass both ASF (with or without dairy) and legumes (with or without nuts). Four countries have food groupings and key messages such that no ASF is technically required (i.e., a single protein group that includes legumes and dairy, no separate dairy group, and no key message about ASF); in most of the rest, meeting dietary guidelines would be possible through vegetarian (i.e., including milk and eggs) but not vegan diets.

### Legumes and nuts

Legumes and nuts are often included in key messages with other protein foods, but they are also recommended as a unique dietary component and, less commonly, included in key messages related to vegetables or fats (**Table 7**). There are key messages concerning legumes and/or nuts in 58% of countries. An additional 10% of countries have no key message about legumes or nuts per se, but imply encouragement to consume them via a message about consuming all food groups on the food guide, which includes legumes and sometimes nuts. Countries are much more likely to include key messages about legumes (56%) than about nuts (19%). Only 3 countries have key messages recommending “moderate” consumption of legumes together with other protein foods (Malaysia, Belgium, and Malta); all the other key messages encourage consumption of legumes. WHO guidance includes encouragement to consume both legumes and nuts as part of a healthy diet (20), but only 12% of countries include a similar positive message about both.

**TABLE 7:** Key messages about legumes and nuts conveyed by more than 5 countries—types and examples

	Number^1^	% (of 90)
Any key message that includes legumes	50	55.6
Any key message that includes nuts	17	18.9
Key message about legumes as a unique dietary component	24	26.7
“Eat dry beans, split peas, lentils and soya regularly” (South Africa)	—	—
“Enjoy a wide variety of nutritious foods from these five groups every day: … plenty of vegetables, including different types and colours, and legumes/beans” (Australia)	—	—
Legumes in protein food message together with animal-source foods	23	25.6
“Eat pulses, fish, poultry, eggs and a little meat regularly” (Nepal)	—	—
Nuts mentioned in protein food message	5	5.6
“A healthy eating pattern includes … A variety of protein foods, including seafood, lean meats and poultry, eggs, legumes (beans and peas), and nuts, seeds, and soy products” (United States)	—	—
Legumes or nuts in a vegetable or fat message	6	6.7
“Eat five servings of vegetables, legumes and fruits every day. The ideal would be to eat three servings of vegetables and/or legumes and two servings of fruit” (Austria)	—	—
“Use good fats, such as unsaturated fatty acids (olive oil), omega-6 (sunflower oil and soya oil) and omega-3 (nuts and soya oil and fatty fish)” (Spain)	—	—
Legumes (soy) mentioned in a message with dairy	6	6.7
“Consume milk, beans, or dairy or soybean products every day” (China)	—	—
Eat legumes daily	14	15.6
“Eat beans and tortillas every day” (Guatemala)	—	—
Eat legumes X times a week (X = several times, 2, 3, 4, or >4)	9	10.0
“Eat pulses (peas, beans and lentils) at least 4 times a week” (Seychelles)	—	—
Substitution message^2^	6	6.7
“Have meat alternatives such as beans, lentils and tofu often” (Canada)	—	—
Not mentioned but implied in diversity message (by reference to food guide)^3^	10	11.1
“Eat foods from each food group every day to have a complete diet” (Portugal)	—	—
No key message conveyed about legumes	40	40.4
No key message conveyed about nuts	73	81.1

^1^Sums to more than 90 because some countries have multiple messages related to legumes and nuts. Also some key messages contain more than 1 idea and are counted for both (e.g., “Eat beans and tortillas every day” is counted both as a “Legumes as a unique dietary component” and an “Eat daily” message).

^2^There are diverse substitution messages; for example, eat legumes instead of meat, or instead of “fatty meat”; eat nuts instead of unhealthy snacks, etc.

^3^Considered to be implied when a key messages directs the reader to eat all food groups, and legumes, or less commonly legumes and nuts, are a separate food group in the food guide.

At present globally there is great diversity in how legumes and nuts are presented in food guides (**Table 8**). Legumes (with or without nuts) are a separate food group in 26% of countries. There is also regional variation in how legumes are grouped; they are most likely to be grouped with “protein foods” in North America (both of 2 countries), the Asia Pacific region (73% of countries), and in Europe (74%), whereas they are not usually grouped with ASF in LAC (8%). Legumes are more likely to be grouped with vegetables in Europe (30% of countries); outside of Europe, only 3 countries group legumes with vegetables. A few countries explicitly place legumes in 2 groups, most commonly in a “protein foods” group and in the vegetables group. Legumes (or legumes and nuts) are most likely to be given their own group in the Near East (50%) and in LAC (54%), reflecting the importance of legumes in the diet. LAC is also the only region where legumes are commonly grouped with starchy staples (33%).

**TABLE 8:** Grouping of legumes and nuts in food guides^1^

	Number	% (of 78)^2^
Legumes grouped with flesh foods or all animal-source foods (with/without nuts)	38	48.7
Legumes grouped with starchy staples	12	15.4
Legumes (pulses, beans, peas) own group	14	17.9
Legumes grouped with vegetables	11	14.1
Legumes and nuts grouped (own group)	6	7.7
Nuts grouped with flesh foods or all animal-source foods (with/without legumes)	28	35.9
Nuts grouped with fats/oils	18	23.1
Could not determine how legumes were grouped	3	3.8
Could not determine how nuts were grouped	22	28.2

^1^Groupings with frequency of ≥5 countries; in addition, there are idiosyncratic groupings by single countries, not shown here. The percentage sums to >100 for legumes because they are sometimes pictured or classified in more than 1 group.

^2^Denominator is the 78 countries that use a food guide and provided it to the FAO.

Regional variation in how nuts are grouped was less apparent, in part because for many countries nuts are not clearly depicted in food guides nor mentioned in brief guides or other available descriptions of food groupings. Whereas it was not possible to determine where legumes are grouped for only 4% of countries globally, this was much more common for nuts, with 28% of countries having no clear depiction or mention of nuts. Globally, there are very mixed messages on nuts, with some countries grouping them with fats/oils and conveying implicit messages on moderation through the proportionality of the graphic, and other countries encouraging consumption, or encouraging consumption of nuts in place of other snacks, or nut oils in place of other fats.

### Dairy

Dairy messages are, in most cases, distinct from other “protein foods” messages. This is the case in 59% of countries, which have a key message about dairy alone, which typically includes milk and milk products (**Table 9**) (63% of countries include dairy foods in any key message). In food guides, dairy is its own food group in 64% of countries, and grouped with protein foods in 31% of countries, whereas only 3 countries (4%) have no visual representation of dairy, and in 1 country (China), dairy and soy are grouped. Considering key messages and food guides together, 75% of countries include dairy in their FBDG.

**TABLE 9:** Key messages about dairy conveyed by more than 5 countries—types and examples

	Number^1^	% (of 90)
Dairy key message	53	58.9
“Consume more milk and milk products” (Estonia)	—	—
“Limit your daily consumption of cheese to one or two slices. Do not drink more than 3–4 glasses of skimmed or semi-skimmed milk or soy products” (Belgium)	—	—
Includes dairy alternatives to fluid milk (cheese, yogurt)	46	51.1
“Consume milk, yogurt or cheese, preferably low-fat, every day” (Argentina)	—	—
Includes nondairy alternatives to fluid milk	10	11.1
“Have some dairy or dairy alternatives (such as soya drinks); choosing lower fat and lower sugar options” (United Kingdom)	—	—
Includes eggs	3	3.3
“Include in your daily diet at least one of the following foods: milk, cheese, curd, cottage cheese or eggs” (El Salvador)	—	—
Consume daily	27	30.0
“Eat milk and dairy products every day” (Spain)	—	—
Quantitative recommendation	13	14.4
“Eat two servings of dairy products a day” (Greece)	—	—
Encourage low-fat forms of dairy	26	28.9
“Use milk and dairy products that are low in fat” (Slovenia)	—	—
Key message based on calcium	6	6.7
“Consume milk, milk products and other calcium-rich foods, such as small fish and shellfish, every day for healthy bones and teeth” (Philippines)	—	—
Not mentioned but implied in diversity message (by reference to food guide)^1^	3	3.3
“Eat foods from each food group every day to have a complete diet” (Portugal)	—	—
No key message conveyed but dairy group is shown in food guide	15	16.7

^1^Considered to be implied when a key message directs the reader to eat all food groups, and dairy is a separate food group in the food guide.

All dairy messages include mention of milk; 51% (comprising 46 of the 53 countries with dairy messages) include “milk products,” yogurt, or cheese in addition to fluid milk; 11% (10 countries, distributed across various regions) include nondairy alternatives to milk such as soymilk or other calcium-rich foods; and in only 3 countries, all in LAC, does the dairy message also include eggs.

Over half of the countries with dairy messages recommend dairy consumption “daily” (27 of 51 countries). A quantitative message is conveyed in key messages of 14% of countries (*n* = 13, of which 8 are in Europe), recommending a mean of 2.4 servings per day (mode = 2); 3 of those countries recommend a specific volume of consumption, of 500 mL per day.

Half of countries’ dairy messages include a mention of “low fat” (29% of all countries, *n* = 26), whereas low-salt and low-sugar dairy are mentioned by only 4 and 5 countries, respectively. Six countries include calcium in their key message about dairy. Four countries guide consumers to consume “more” dairy, whereas 2 countries include guidance to limit or moderate dairy consumption.

There are significant regional differences in dairy representation and messages. Dairy is strongly emphasized in North America, the Near East, and Europe, where it is depicted as a separate food group in nearly all countries (100%, 100%, and 85%, respectively), and where most countries have a key message about dairy (100%, 75%, and 82%, respectively). In contrast, fewer countries in Africa, Asia Pacific, and LAC show dairy as a separate food group (50%, 60%, and 38%, respectively) or have a key message about dairy (57%, 53%, and 30%, respectively); the 3 countries without visual representation of dairy in any food group are in those regions as well (Sierra Leone, Vietnam, and Belize).

### Fats and oils

Most countries (89%) have a key message on limiting fat. Fewer than half of all countries (44%) have a message on the quality of fats apart from limiting consumption. A minority (18%) of countries include key messages on healthy fats that should be consumed regularly, advice found only in North America (100%), Europe (33%), and 3 countries in Latin America (11%) (e.g., “Use olive oil as the main added lipid”: Greece).

The WHO Healthy Diet Fact Sheet (20) advises that “unsaturated fats (e.g., found in fish, avocado, nuts, sunflower, canola and olive oils) are preferable to saturated fats (e.g. found in fatty meat, butter, palm and coconut oil, cream, cheese, ghee and lard).” This advice is echoed by 29% of countries having key messages that indicate preference for unsaturated over saturated fats (e.g., “Limit intake of solid fats and replace with vegetable oils”: Lebanon).

In the food guides of 35% of countries, healthy and unhealthy fats are grouped together in the same “fat” group, and in 36% of countries there is a mixed or double visual message about the fat group. For example, the food guide might include clear examples of healthy fats (e.g., “olive oil” or “sunflower oil”), and also say “use sparingly.” Or, healthy fats can be contained in a small slice of a circle graphic, similar to sweets, which are often also contained in an identically small, separate slice of a circle graphic. Some countries contain words on the graphic next to the fat group, to explain this dual-purpose graphical representation; for example, “Oils and spreads: Choose unsaturated oils and use in small amounts” (United Kingdom). Twenty-three percent of fat graphics include nuts, seeds, or peanut butter, and 24% include avocado or coconut.

In the food guides of 18% of countries (not necessarily the same 18% with key messages about healthy fats), there is an explicit “healthy fats” group, which is clearly depicted as healthy by both the type of fat and its placement (such as a larger portion, or a yellow “traffic-light” indication). However, 33% of all food guides indicate the fat group as limiting, either with a red “traffic light” symbol, or by combining fats with sweets and other “junk foods.” Eight percent of countries do not include fats in their food guides at all (e.g., the United States), despite some of those countries (including the United States) having key messages on consuming healthy fats.

### Foods and food components to limit

All countries have at least 1 key message to limit certain types of foods or components of foods. Several countries focus the majority of their key messages on types of foods to limit, as a complement to the food guide (e.g., “Limit foods and drinks from the Top Shelf of Food Pyramid. This is the most important Healthy Eating Guideline, as these are high in fat, sugar and salt”: Ireland).

The most common messages involve limiting salt, fat, and sugar. Ninety percent of countries have a key message about limiting salt; 89% have a key message about limiting fat of some kind; and 84% have a message about limiting sugar; 70% of countries have a message about all 3. Considering food guides and key messages together, salt, sugar, and fat are each cautioned against in over 90% of countries. The fourth most common type of “limit” message is about highly processed foods, which are advised against in 28% of countries (e.g., “Consume less carbonated beverages and artificial juices because they damage your health”: Paraguay). Messages about limiting highly processed foods appear in all regions except North America, and are somewhat more common in LAC (44% of LAC countries). Nearly one-quarter of countries globally (23%) have a message to limit or moderate consumption of meat of some kind; 13% concern meat in general, and 11% are specifically about red, processed, and/or cured meats. Such messages to limit meat consumption are absent in North America and Africa. In food guides, some European countries also identify red meats and/or processed meats in the tip of the pyramid, conveying a message of moderation in consumption of these foods. Other specific foods to limit are mentioned much more infrequently: animal foods in 3 countries, eggs specifically in 2 countries, and refined grains in 2 countries. A small minority of countries (8%) cite metabolic factors or noncommunicable diseases as explicit reasons for limiting the types of foods mentioned.

The WHO Healthy Diet Fact Sheet recommends “less than 5 g of salt per day and use iodized salt” (20). Whereas 89% of countries have a message consistent with the WHO recommendation to limit salt (sodium) consumption from all sources, only 8 countries have a quantitative message about salt intake limits consistent with the WHO ceiling of 5 g/d. A message to use iodized salt is included by 18% of countries.

WHO recommends “less than 10% of total energy intake from free sugars” (20). Limiting the intake of sugars generally is advised by 84% of countries; only 1 country (the United States) includes a quantitative message on limiting sugar (<10% of calories from added sugars, consistent with WHO guidelines). Key messages by 46% of countries mention a need to limit sugar-sweetened beverages specifically.

WHO recommends “less than 30% of total energy intake from fats; unsaturated fats...are preferable to saturated fats; industrial *trans* fats are not part of a healthy diet” (20). Guidance to limit at least some type of fat is given by 89% of countries: 53% of countries’ key messages are clearly interpretable as limiting intake of *total* fats; only 2 countries (Albania and Mongolia) set a quantitative limit on total fat consumption, of <30%; 43% of countries have messages about limiting saturated fat; and 13% of countries advise limiting *trans* fats (or “margarine”). By way of providing further guidance, 22% of countries suggest cooking methods to reduce fat, and 29% suggest healthier alternatives to saturated fat. An example of the simplest kind of message is: “Limit sugar, salt and fat” (Qatar). An example of a comprehensive and complex message is:

“(a) Less red and processed meat—Eat less red and processed meat, no more than 500 grams a week. Only a small amount of this should be processed meat. (b) Less salt—Choose food with less salt. Use less salt when you cook, but choose salt with iodine when you do use it. (c) Less sugar—Hold back on the sweets, pastries, ice creams and other products containing lots of sugar. Cut back on sweet drinks in particular”

(Sweden) (20).

### Alignment with WHO guidance

**Table 10** shows the proportion of countries where key messages and/or food guides align with recommendations in the WHO Healthy Diet Fact Sheet (20). Considering both key messages and the food guide, the majority of countries’ FBDG align with the following WHO guidance (20): consuming fruits and vegetables (100%), and a quantitative target of 5 portions of fruits and vegetables a day (51%); consuming legumes (96%); consuming whole grains (53%); and limiting free sugars (94%), salt (91%), and fat (94%). Fewer countries reflect other elements of the WHO guidance (20), including guidance to consume nuts (36%), and to use iodized salt (18%). Although WHO guidance does not mention ASF, it is important to recognize the ubiquity of ASF in FBDG—which could reflect country-level assessment of the best path to meet micronutrient needs given cultural dietary patterns.

**TABLE 10:** Percentage of countries where key messages, food guides, or brief consumer guidance align with WHO messages (20)

WHO Healthy Diet Fact Sheet recommendations for a healthy diet	Message in national FBDG	Percent of countries that have this key message (%)	Have a key message and/or include in food guide (%)
Fruits, vegetables, legumes (e.g., lentils, beans), nuts and whole grains (e.g., unprocessed maize, millet, oats, wheat, brown rice)	A healthy diet contains: fruits, vegetables	93	100
	A healthy diet contains: legumes	56	96
	A healthy diet contains: nuts	19	36
	A healthy diet contains: whole grains	44	53
At least 400 g (5 portions) of fruits and vegetables per day.	400 g (5 portions) of fruits and vegetables per day	33	51
<10% of total energy intake from free sugars.	Limit sugar	84	94
<30% of total energy intake from fats. Unsaturated fats (e.g., found in fish, avocado, nuts, sunflower, canola, and olive oils) are preferable to saturated fats (e.g., found in fatty meat, butter, palm and coconut oil, cream, cheese, ghee, and lard). Industrial *trans* fats (found in processed food, fast food, snack food, fried food, frozen pizza, pies, cookies, margarines, and spreads) are not part of a healthy diet	Limit fat	89	94
	Prefer unsaturated fat to saturated fat	29	n/a
	No *trans* fat	13	n/a
<5 g of salt (equivalent to $\tilde{1}$ teaspoon) per day and use iodized salt	Limit salt	90	91
	Use iodized salt	18	18

^1^FDBG, food-based dietary guidelines; n/a, not applicable.

## Discussion

There is a high level of consistency across countries globally on several dietary recommendations, including: consumption of a diversity of foods; consumption of abundant fruits and vegetables (≥5 servings or 400 g); inclusion of starchy staples, ASF, and legumes in the diet; and avoidance of excessive salt, sugar, and fat. Almost all food guides indicate proportionality in the diet, with the largest shares in starchy staples and fruits and vegetables. Other areas are less consistent across countries. The most important to discuss are dairy, meat, fats, and nuts.

Dairy is most commonly conveyed as its own food group (by 64% of countries), and 60% have key messages about dairy. That global majority, however, is driven by Europe and North America. A significant minority of countries do not include a separate dairy group—usually because dairy products are included in a general protein group as alternative protein sources—which leaves open the question of whether dairy is considered by country authorities to be a daily requirement for all adults in all regions, including those regions where lactose intolerance is prevalent in adulthood. The exact incidence of lactose intolerance is unknown, but according to some estimates the relative or absolute absence of lactase occurs in 70% of the world's population, particularly in Asia, Africa, and South America (26). Key messages on dairy, and separate dairy groups in food guides, are markedly less common in those regions than in North America, Europe, and the Near East. Food guides and key messages generally include alternatives to fluid milk such as hard cheese, which render the dairy group somewhat more feasible for individuals who cannot digest lactose. A dairy group might not be included in countries where dairy products are not part of the food culture.

Meat, particularly red meat, is treated differently across countries. Although a few countries describe meat as nonsubstitutable (all in LAC), 23% recommend limits on meat intake, most commonly in Europe. These recommendations seem to contrast, but meat consumption can have different nutritional and environmental consequences depending on the type of meat and level of intake (27), and countries’ key messages might relate to where the majority of their populations stand with regard to typical levels of meat consumption. In some populations, like in Guatemala where meat is consumed at low levels among the most nutritionally vulnerable, the purpose of the recommendation to include meat consumption is to avoid anemia (28). This is in contrast to the Netherlands, for example, where in the majority of the population meat is consumed at high levels that can exceed limits recommended by international organizations (29, 30) and that pose risks to public health and environmental sustainability (31). In countries where there is a significant segment of the population vulnerable to undernutrition, it is important that recommendations for meat are presented in terms of moderate consumption, to guard against a swing from low consumption to excessive consumption, as is sometimes observed in the nutrition transition. Equity and economic concerns are also important: Benin provides an example of a guideline about meat that explicitly acknowledges that not everyone might be able to access meat often, and provides an alternative way to satisfy the dietary recommendation: “When there is no meat, fish or eggs in a given day, you can replace them with pulses, peanuts, soybeans, soya, cheese or peas. All these foods are rich sources of protein” (Benin).

Are fats and oils a recommended food group, or merely to be limited? In food guides, the message consumers are meant to take away is in many cases unclear—perhaps more unclear than any other type of message. Most countries have a key message to limit certain kinds of fats or total fat, but important nuance about type and quality appears difficult to convey in food guide graphics, which often depict healthy and unhealthy fats in the same group, often with the visual suggestion of reducing consumption. Countries with recently updated guidance have made shifts toward differentiating types of fats and recommending healthy fats, such as in Argentina, Uruguay, and the United Kingdom.

Relatedly, the placement of nuts and seeds is also mixed. Nuts and seeds are recommended as positive in a protein food group in 36% of countries, but are grouped with fats in 23% of countries. In the fats group, these foods suffer from the same lack of clarity (is the message to limit or encourage? ) that emerges generally from representations of fat groups. This is important because consumption of nuts is recommended by WHO (20), and if countries imply they are unhealthy by inclusion with other foods to avoid or limit (such as fats in general), then national guidelines could be in opposition to global guidelines.

### Future frontiers in FBDG

According to other analyses that have focused on sustainability in dietary guidelines, sustainability of diets is not addressed in most current FBDG, although it is an important issue that is likely to gain traction. Gonzalez Fischer and Garnett (17) found that only 4 countries explicitly include sustainability in their FBDG, with an additional 4 having quasi-official sustainable diets guidelines, and at least 2 more having discussed sustainability in the process of developing FBDG. Incorporating sustainability into guidelines often involves rethinking protein group recommendations away from meat, particularly red meat (17, 32, 33). Several countries (mainly in Europe) have done that, although it is not clear whether red meat limitation messages are motivated by concerns for health, sustainability, or both; the motivation for recommendations was outside the scope of this review. The general emphasis in key messages on fish as an important or even nonsubstitutable food (in 17% of countries) could also need consideration from a sustainability standpoint. How to feed 9 billion people sustainably—requiring provision of foods to nourish without depleting natural resources—remains a pressing question for dietary recommendations globally (34).

Although all FBDG incorporate sociocultural factors to some extent, greater attention in some FBDG could be paid to socioeconomic equity, inclusion of indigenous groups (e.g., through food examples commonly consumed), and greater attention to the nutrition transition and the rise in consumption of ultraprocessed or “junk” foods. Fewer countries (just over one-quarter) recommend limits on highly processed foods, but that guidance is more common in newer FBDG, particularly in LAC. For example, Uruguay's FBDG revision focuses on “ultra-processed foods” as a category of foods to limit (35).

Proportionality is almost always suggested in FBDG, but in many countries is difficult to operationalize in exact terms. In food guides, proportionality is suggested as a pie chart in circle graphics (see Malta's food guide, Figure 1) and in other types of graphics. A small number of countries have recently relinquished a graphical approach altogether: Brazil's guidelines emphasize consumption of minimally processed and natural foods, and home-cooked meals. Sweden and Denmark are 2 countries that, in line with WHO guidance (20), focus simply on key foods to eat more of or less of, rather than giving a comprehensive description of a daily diet (see Sweden's food guide, Figure 2). A minority of countries convey servings or gram amounts in their key messages or food guides, and most often for fruits and vegetables—probably because WHO identifies a recommended minimum amount of fruits and vegetables per day (400 g) (20). Clear proportions and quantities can be very helpful, however, for the purpose of monitoring cost or consumption of recommended diets.

Regional guidelines could be a stepping stone between global and national FBDG in terms of facilitating both the FBDG process and comparisons of the cost or consumption of recommended diets across countries. There are some notable similarities across countries within the same region; for example, the way protein foods are grouped in Latin America (2 groups: all ASF, legumes) as opposed to their groupings in North America (2 groups: animal and plant proteins, dairy), or the emphasis on healthy fats in Europe. Some regions have already gone in the direction of regionally aligned recommendations, including the Nordic countries, and to some extent LAC where many countries worked with regional agencies (Instituto de Nutrición de Centroamérica y Panamá and Caribbean Food and Nutrition Institute) to develop their guidance. A recent publication summarized all South Asia national FBDG in 1 regional amalgam, for the purpose of comparing the cost of meeting FBDG across countries, and found that recommendations were largely similar across countries in the South Asia region, with 2 even using the same food pyramid graphic (36). In West Africa it could be fruitful to explore FBDG development and alignment by latitude, because food cultures may be more consistent across latitudes than within national borders.

### Conclusions

The question of “what is a healthy diet?” is pressing, at a time when poor-quality diets are increasingly recognized for their large contribution to malnutrition in all its forms (37). It is helpful to understand which elements of diet are commonly, or even universally, considered important for diet quality by country authorities responsible for FBDG. This review has concluded that there is some relatively simple guidance common to most FBDG: to consume fruits and vegetables and starchy staples as the bulk of the diet; to include ASF and legumes; to limit salt, sugar, and fat; and to consume a diversity of types of food in appropriate proportions. Largely, these most common messages align with WHO guidance, except that WHO also recommends consuming nuts and whole grains, differentiates between types of fat, and does not include guidance about ASF (20). Which ASF to consume preferentially, and in what amounts, is not resolved globally or across countries, neither with regard to dietary necessity or environmental sustainability. Clearer parameters on ASF (including dairy, eggs, meat, and fish) and fats/oils are needed from the global-authority level, for countries to adopt and adapt to their food cultures. FBDG development and revision warrants increased attention to ecological impacts of diets and guidance incorporating sustainability; and enhanced handling of sociocultural factors including economic disparities, rapid dietary transitions toward junk/ultraprocessed food consumption, and differences in dietary patterns of social minority groups such as indigenous peoples. At the same time as FBDG are better tailored to individual populations, further global recommendations around healthy and sustainable diets would be helpful for use and adaptation in country-level FBDG, and for monitoring key aspects of diet quality across countries and globally.

## Notes

Funds for the writing of this article were provided by the UC Davis World Food Center; the FAO Nutrition and Food Systems Division; and through a grant provided by the Bill & Melinda Gates Foundation to *Intake* - Center for Dietary Assessment, FHI 360. During the early stages of preparation both financial and technical support were provided by the FAO Nutrition and Food Systems Division.

Author disclosures: AH, MA, CA-S, JC, KC, and EM: no conflicts of interest.

Abbreviations used: ASF, animal-source food; FBDG, food-based dietary guideline; LAC, Latin America and the Caribbean; PAHO, Pan American Health Organization.
